# Advanced X-ray polarimeter design for nuclear resonant scattering

**DOI:** 10.1107/S1600577520015295

**Published:** 2021-01-01

**Authors:** Berit Marx-Glowna, Ingo Uschmann, Kai S. Schulze, Heike Marschner, Hans-Christian Wille, Kai Schlage, Thomas Stöhlker, Ralf Röhlsberger, Gerhard G. Paulus

**Affiliations:** a Helmholtz-Institut Jena, Fröbelstieg 3, D-07743 Jena, Germany; b Institut für Optik und Quantenelektronik, Friedrich-Schiller-Universität Jena, Max-Wien-Platz 1, D-07743 Jena, Germany; c Deutsches Elektronen-Synchrotron DESY, Notkestrasse 85, D-22607 Hamburg, Germany

**Keywords:** X-ray polarimeter, nuclear resonant scattering, asymmetric reflection, channel-cut crystal

## Abstract

This work presents the improvements in the design and testing of polarimeters based on channel-cut crystals for nuclear resonant scattering experiments at the 14.4 keV resonance of ^57^Fe. By using four asymmetric reflections at asymmetry angles of α_1_ = −28°, α_2_ = 28°, α_3_ = −28° and α_4_ = 28°, the degree of polarization purity could be improved to 2.2 × 10^−9^. For users, an advanced polarimeter without beam offset is now available at beamline P01 of the storage ring PETRA III.

## Abstract 2

An advanced X-ray polarimeter for nuclear resonant scattering experiments, which is now available for users at beamline P01 of the storage ring PETRA III, is reported. The new polarimeter provides a hitherto unachieved degree of polarization purity of 2 × 10^−9^ for the nuclear resonance of ^57^Fe.

## 1. Introduction   

**Figure 1:**
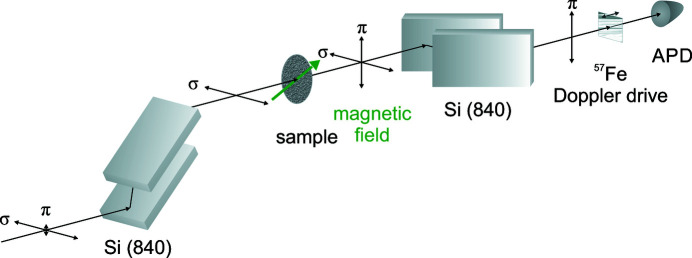
Schematic setup for nuclear resonant scattering with the polarization filtering method. The incoming radiation from the left is polarized by the first channel-cut crystal. Subsequently, the beam impinges on the magnetically anisotropic sample under investigation. The green arrow indicates the direction of the external magnetic field that induces optical activity via X-ray magnetic linear dichroism. The analyzer crystal in the crossed setting transmits only the photons which have undergone nuclear resonant σ- to π-scattering.

Nuclear resonant scattering of synchrotron radiation (NRS) is an established methodology ranging from the study of structure and dynamics of condensed matter (Gerdau & de Waard, 1999 ▸, 2000 ▸; Röhlsberger, 2004 ▸) to the realization of quantum optical phenomena (Siddons *et al.*, 1995 ▸; Toellner *et al.*, 1995 ▸; Röhlsberger *et al.*, 2010 ▸, 2012 ▸; Schulze, 2018 ▸). In 1974, Ruby discussed the idea to use synchrotron radiation as source for Mössbauer measurements for the first time (Ruby, 1974 ▸). The advantages of synchrotron radiation in comparison with radioactive sources are the much higher brilliance, energy tuneability, time structure and linear polarization of the pulsed beam. The first experiment demonstrating nuclear resonant of synchrotron radiation took place in 1985 at the storage ring DORIS (DESY, Hamburg) employing the 14.4 keV resonance of ^57^Fe (Gerdau *et al.*, 1985 ▸). Nuclear resonances have a very narrow energy bandwidth of typically 10^−7^ to 10^−11^ eV, in the case of ^57^Fe the natural linewidth is 4.7 neV. The challenge of this method is to discriminate the small fraction of nuclear resonant scattered photons against the energetically broad synchrotron beam. NRS beamlines at synchrotrons of the third generation like P01 at PETRA III provide 7 × 10^13^ photons s^−1^ within an energy bandwidth of around 1 eV after double-crystal silicon high-heat-load monochromators. There are different techniques to discriminate the resonantly scattered photons against the nonresonant background, which are often used in combination. Usually, the time delay of the scattered photons due to the nuclear lifetime is exploited by time filtering. Today, this is done by using avalanche photodiodes (Kishimoto, 1992 ▸; Baron, 2000 ▸). In the higher energy range from 15 to 50 keV it is done with plastic scintillators (Metge *et al.*, 1990 ▸). The time filtering method alone is not adequate for discrimination and has to be combined with an improvement of the signal-to-noise ratio, in general by high-resolution monochromators (Faigel *et al.*, 1987 ▸; Toellner, 2000 ▸; Shvyd’ko, 2013 ▸). Other methods which are used to suppress the nonresonant background are: grazing-incidence anti-reflection (GIAR) films (Grote *et al.*, 1991 ▸; Alp *et al.*, 1993 ▸), multilayer structures (Trammell & Hannon, 1978 ▸; Röhlsberger *et al.*, 1993 ▸), high-speed shutters (Toellner *et al.*, 2011 ▸), the nuclear lighthouse effect (Röhlsberger *et al.*, 2001 ▸) and polarization filtering (Toellner *et al.*, 1995 ▸; Alp *et al.*, 2000 ▸; Siddons *et al.*, 1995 ▸). The last one is a technique that makes use of the strong optical activity that is inherent to the nuclear scattering process. In this technique an X-ray polarimeter is used to filter the resonantly scattered photons that have undergone a change in their polarization state with respect to the white beam, if the nuclei are exposed to a magnetic field (see Fig. 1 ▸).

Hitherto X-ray polarimeters for NRS are based on Bragg diffraction in channel-cut crystals with Bragg angles near the Brewster angle for X-rays of 45° (Ramaseshan & Ramachandran, 1954 ▸; Hart, 1978 ▸). For the nuclear resonance of ^57^Fe at 14.413 keV, typically silicon (840) channel-cut crystals with two asymmetric reflections and a Bragg angle of θ_B_ = 45.1° are used. Polarization purities of δ_0_ = 6 × 10^−7^ (Toellner *et al.*, 1995 ▸) and δ_0_ = 4 × 10^−8^ (Röhlsberger *et al.*, 1997 ▸) were achieved with such polarimeters. The degree of polarization purity δ_0_ is defined as the ratio of the transmission of the π-component to the transmission of the σ-component (Alp *et al.*, 2000 ▸).

For the equipment of a nuclear resonance beamline with a zero beam-offset polarimeter, we have improved these values by using four reflection channel-cut crystals with smaller asymmetry angles. These improvements enable the polarimeter to be used as a user instrument. The corresponding design and its performance are discussed in this article.

## 2. Design   

The following design study is optimized for the high-resolution dynamics beamline P01 at PETRA III (DESY, Hamburg, Germany). The beamline provides an energy bandwidth of 0.4 eV at 14.41 keV employing a cryogenically cooled high-heat-load double-crystal Si (311) monochromator. The divergence of the beam (FWHM) amounts to 9 µrad vertical and 17 µrad horizontal. At the installation point of the polarimeter the beam size is 0.8 mm in the vertical and 2.5 mm in the horizontal.

The polarimeter design has to provide sufficient photon flux for the experiments on the one hand and a good polarization purity to suppress the nonresonant background on the other. Considering the ratio of the energy bandwidth of the polarizer and the natural line width of the ^57^Fe-isotope, the upper limit for the degree of polarization purity of the polarimeter has to be ideally better than 1 × 10^−8^. In addition for a permanent installation of the polarimeter there should be no beam offset after the polarizer. This enables a convenient swap-in/swap-out mode of operation with a minimum of adjustment needed for the instruments downstream of the polarizer.

To make use of the full X-ray beam delivered by the undulator, the angular acceptance of the polarizer crystal should match the divergence of the incident beam. This is not easy because the intrinsic Darwin width of 45° Bragg reflections in the regime of hard X-rays are in the range of a few microradian, but there are two options to achieve this. On the one hand, one can use collimating lenses to decrease the divergence; on the other, it is possible to enhance the angular acceptance of the polarizer by using asymmetric reflections. Asymmetric reflection means that the diffracting lattice planes are not parallel to the crystal surface. The asymmetry parameter *b*, where
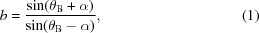
is used to quantify the asymmetry, where θ_B_ is the Bragg angle and α is the asymmetry angle which defines the angle between the crystal surface and the diffracting lattice planes. By definition, α is counted negative if the incidence angle relative to the crystal surface is smaller than the exit angle. Under the condition of asymmetric reflection the angular width of the rocking curve *D* for a symmetric reflection changes to *D*_−_, 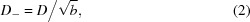
on the input side of the crystal. The more negative the angle of asymmetry α is, the larger the angular acceptance will be. This is accompanied by a broadening of the geometric beam width *S*_+_, where 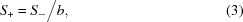
at the output side and a decrease of the angular divergence of the exit beam, so that the product of beam size and angular divergence stays constant. *S*_−_ denotes the beam size at the entrance side. Another advantage of asymmetric reflections is the improvement in the degree of polarization purity.

To polarize the radiation, we use Bragg reflections close to the Brewster angle of 45°. Silicon (840) provides an Bragg angle of θ_B_ = 45.1° for the nuclear resonance of ^57^Fe. The deviation of 0.1° results in a polarization purity of 1.1 × 10^−4^ for a single symmetric reflection.

By increasing the number of reflections *n* inside the channel-cut crystal, δ_0_ can be strongly enhanced. However, with increasing number of reflections, the diffracted intensity and angular acceptance decreases. So the combination of asymmetric reflections with channel-cut crystals can increase the polarization purity while maintaining angular acceptance. However, the increase in asymmetry with a simultaneous increase in the number of reflections leads geometrically to an enlargement in crystal size. Accordingly the number of reflections inside the channel-cut crystal and the size of asymmetry is limited. Table 1 ▸ gives an overview of the design parameters for different asymmetry angles and number of channel-cut reflections.

In order to achieve a polarization purity of better than 10^−8^, channel-cuts with four reflections each should be used. The degree of asymmetry of −28° chosen by us represents a compromise between photon intensity and crystal size. This will lead to different efficiencies for polarizer and analyzer, due to the different source divergence values in the horizontal and vertical direction at beamline P01. An adjustment of the efficiencies as well as a full utilization of the divergence of the beamline can be achieved by combining the polarimeter with collimating X-ray lenses after the monochromator, also at this asymmetrie angles. The throughput of the polarimeter in combination with lenses is then 0.46 in parallel position. The new ultra-low-emittances sources (APS-U, PETRA IV) will improve the efficiency of the polarimeter, due to the better horizontal divergence.

The advantage compared with the −43° system is, besides the improvement of the degree of polarization purity, also an improvement of the transmitted photon intensity, as well as a smaller asymmetry angle which keeps the size of the channel-cut compact. In addition, the smaller asymmetry leads to a greater penetration depth of the radiation, which has less requirements on the processing of the surfaces, and relieves the adjustment of the channel-cut crystals.

**Figure 2:**
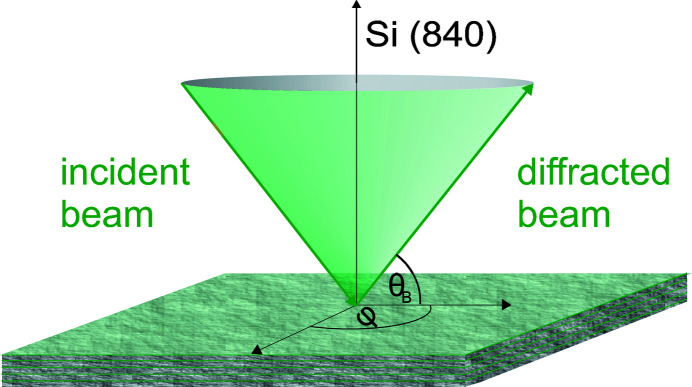
Kossel cone of Si (840) for θ_B_ = 45.1°. The azimuthal angle φ has to be carefully aligned to achieve a high polarization purity δ_0_.

**Figure 3:**
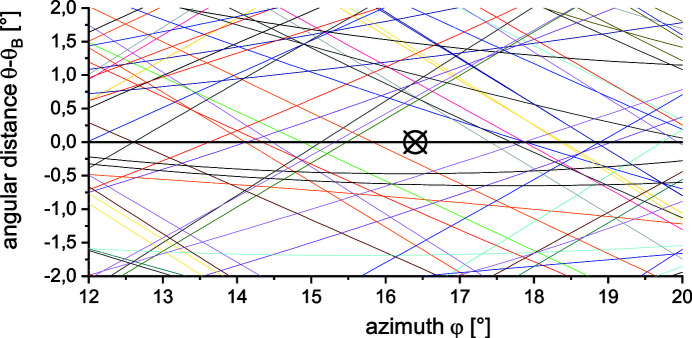
The black cross illustrates the chosen azimuthal orientation of the polarimeter crystals. The coloured lines show the angular distance of the incident vector to Bragg angles of other crystal lattice planes for the first reflection. The azimuth angle φ = 0° corresponds to (1 3 0).

Another important aspect of polarimeter design is indispensable to achieve high polarization purity levels: this is the azimuthal orientation of the channel-cut crystals to avoid multiple-beam cases (Marx *et al.*, 2013 ▸). To minimize their influence, there is the possibility to rotate the crystal azimuthally, *i.e.* around an axis that is perpendicular to the diffracting lattice plane (see Fig. 2 ▸) in order to find the azimuthal position with the largest distance to neighbouring Umweg reflections (Renninger, 1937 ▸). We chose an incidence vector of *V*_i_ = (0.33, 0.92, 0.20) on the crystal surface in the Cartesian coordinate system. Figure 3 ▸ shows the dispersion of Umweg reflections in the vicinity of the polarizing Bragg reflection as a function of the azimuthal angle φ. The optimum position is the azimuth with the largest angular distance to Umweg reflections, marked by the cross.

**Figure 4:**
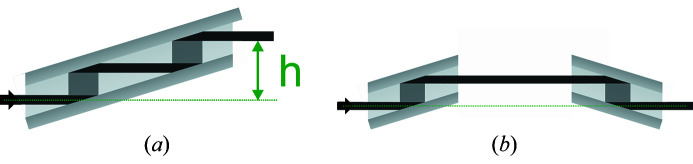
Two four-reflection polarizers in comparison. (*a*) A four-reflection channel-cut with beam offset *h*. (*b*) A quasi-channel-cut consisting of two-reflection channel-cuts in (*n*, +*n*) setting, with zero beam offset.

For a permanent installation of the polarimeter on a beamline it is of great advantage for users if the beam offset *h* of the polarizer is compensated for. This can be achieved by separating the four-reflection channel-cuts of the polarizer into two two-reflection channel-cuts in antiparallel (*n*, +*n*) setting (see Fig. 4 ▸).

**Figure 5:**
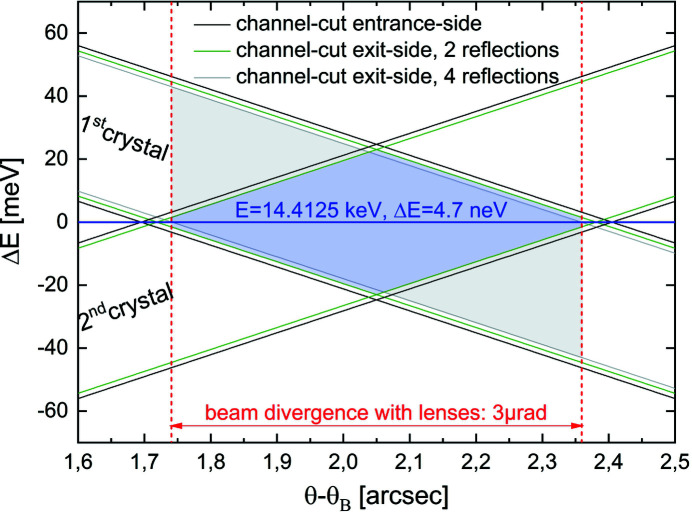
DuMond diagram for two asymmetrically cut two-reflection channel-cut crystals in comparison with one four-reflection channel-cut, as shown in Fig. 4 ▸. The vertical axis shows the detuning of the energy from the nuclear resonance of *E* = 14.4125 keV. Due to the small energy bandwidth of the nuclear resonance, there is no intensity loss in the setting without beam off-set.

The DuMond diagram (DuMond, 1937 ▸) in Fig. 5 ▸ compares the (*E*, θ)-space of the diffracted X-ray radiation for the two polarizer settings. The solid green and grey lines represent plots of the Bragg equation shifted by ±FWHM/2 of the rocking curve for a two-reflection and four-reflection channel-cut. Blue shaded is the diffracted phase-space for the two two-reflection channel-cut crystals in (*n*, +*n*)-setting, grey shaded for the one four-reflection channel-cut crystal. So there is a nearly 50% loss if one wants to use the full energy bandwidth of the Si(311) monochromator of 0.4 eV in combination with lenses. For nuclear resonant scattering experiments at ^57^Fe, however, only an energy bandwidth of 4.7 neV is required, so that the photon intensity within the nuclear bandwidth is the same in both polarizer settings.

## 3. Experiment   

**Figure 6:**
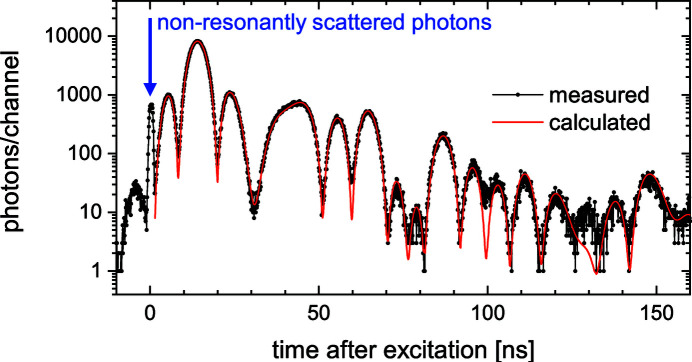
Time spectrum of a 9.3 µm-thick foil of ^57^Fe (95% enrichment) magnetized at 45° to the polarization direction. The red line is a fit to the data via the program package *CONUSS* (Sturhahn, 2000 ▸). The use of the polarimeter makes it possible to fit the time spectrum already 1.5 ns after excitation.

We have characterized the performance of the polarimeter at the high-resolution dynamics beamline P01 at PETRA III in Hamburg. During the measurement, the synchrotron was operating in the 40-bunch filling mode at a storage ring current of 80 mA. The energy of the the Si (311) high-heat-load monochromator beamline was adjusted to the 14.4125 keV resonance of ^57^Fe. As polarizer and analyzer, four-reflection Si (840) channel-cuts with an asymmetry of α_1_ = −28°, α_2_ = 28°, α_3_ = −28° and α_4_ = 28° were used. For the determination of the polarization purity of the polarimeter, the analyzer crystal was rotated from the parallel position to the crossed position. In each position the rocking curve of the Bragg reflection was measured. The photons were detected via photodiode and APD behind the analyzer. The incident photon flux from the polarizer was monitored by an ionization chamber. The measuring time was 0.5 s per measuring point in the parallel position and 1 s per measuring point in the crossed position. The degree of polarization purity is determined by dividing the integrated intensity of the measured rocking curve in the crossed position to the integrated intensity of the rocking curve in the parallel position. This results in a degree of polarization purity of δ_0_ = (2.2 ± 2.0) × 10^−9^. Figure 6 ▸ shows as an example a time spectrum of a 9.3 µm ^57^Fe-enriched film, measured with the designed polarimeter. The total data collection time was 69 min. To maximize the XMLD signal, the film was magnetized 45° to the polarization direction.

The use of the polarimeter allows the suppression of non-resonantly scattered photons, indicated by the blue arrow in Fig. 6 ▸. In contrast to the method with high-resolution monochromator and time discrimination, polarization filtering makes the full spectrum already 1.5 ns after excitation accessible. This will enable access to short-lived isotopes like ^169^Tm and ^187^Os with lifetimes of 5.9 and 3.4 ns, respectively. Moreover, the early times after resonance excitation are highly interesting to reveal the dynamics of cooperative effects like superradiance or the collective Lamb shift in X-ray quantum optics.

## 4. Conclusion   

**Figure 7:**
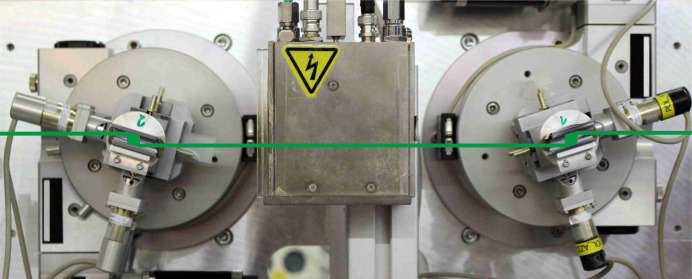
Permanent setup of the polarizer consisting of two channel-cut crystals in Bartels geometry at beamline P01 of PETRA III. The incoming beam from the right is polarized by two two-reflection channel-cuts in (*n*, +*n*)-setting.

We have developed a polarimeter for the 14.4 keV nuclear resonance energy of ^57^Fe that uses four asymmetric reflections each for the polarizer and analyzer. With this polarimeter, we improved the degree of polarization purity by more than one order of magnitude compared with previously published performances of polarimeters working at this energy. The purity of this polarimeter is still limited on the one hand by multiple-beam-cases and on the other hand by the divergence of the beam from the undulator (Schulze, 2018 ▸). As a second feature, the polarizer was designed to be composed of two channel-cuts in Bartels geometry. This allows the polarimeter to be inserted into the beam path without any beam offset. The polarimeter setup was installed as a user instrument for swap-in/swap-out operation at beamline P01 at PETRA III (DESY, Hamburg) (see Fig. 7 ▸).

Besides operation in the forward direction, the analyzer, consisting of one four-reflection channel-cut crystal, can be mounted on the 2θ-arm of a θ–2θ-goniometer. This enables, for example, the polarization-dependent investigation of layer systems in reflection geometry. Recently, this setup was the key for the discovery of several quantum optical effects in the X-ray regime (Heeg *et al.*, 2013 ▸, 2015 ▸; Haber *et al.*, 2016 ▸).

## Figures and Tables

**Table 1:** Calculated polarization purities δ_0_ for different asymmetry angles α, number of reflections *n*, accepted angular width of the incoming beam *D*_−_ and angular acceptance of the channel-cut crystal *D*_+_. *S*_+_ describes the broadening of the geometric beam width and (*I*_max_/*I*_0_)_σ_ the ratio of the diffracted intensity at the rocking curve maximum to the incident intensity. The best X-ray polarimeters for NRS so far use an asymmetry angle of α_1_ = −43° (Röhlsberger *et al.*, 1997 ▸) and *n* = 2 reflections. We chose an asymmetry angle of α_1_ = −28° and *n* = 4 reflections to improve δ_0_

α_1_ (°)	*n*	*D*_−_ (µrad)	*D*_+_ (µrad)	*S*_+_ (mm)	(*I*_max_/*I*_0_)_σ_	δ_0_
0	1	1.9	1.9	2.5	0.95	1.1 × 10^−4^
0	2	1.9	1.8	2.5	0.9	1.6 × 10^−7^
0	4	1.9	1.7	2.5	0.81	5.4 × 10^−13^
−28	1	3.4	3.4	8.1	0.93	9.2 × 10^−5^
−28	2	3.4	3.2	8.1	0.87	1.1 × 10^−7^
−28	4	3.4	3.0	8.1	0.76	2.5 × 10^−13^
−43	1	9.9	9.9	68.1	0.83	4.5 × 10^−5^
−43	2	9.9	9.0	68.1	0.68	1.1 × 10^−8^

## References

[bb2] Alp, E. E., Mooney, T. M., Toellner, T., Sturhahn, W., Witthoff, E., Röhlsberger, R., Gerdau, E., Homma, H. & Kentjana, M. (1993). *Phys. Rev. Lett.* **70**, 3351–3354. 10.1103/PhysRevLett.70.3351 10053846

[bb1] Alp, E., Sturhahn, W. & Toellner, T. (2000). *Hyperfine Interact.* **125**, 45–68.

[bb3] Baron, A. Q. R. (2000). *Hyperfine Interact.* **125**, 29–42.

[bb4] DuMond, J. W. (1937). *Phys. Rev.* **52**, 872–883.

[bb5] Faigel, G., Siddons, D., Hastings, J., Haustein, P., Grover, J., Remeika, J. & Cooper, A. (1987). *Phys. Rev. Lett.* **58**, 2699–2701. 10.1103/PhysRevLett.58.2699 10034822

[bb7] Gerdau, E. & de Waard, H. (1999). Editors. *Nuclear Resonant Scattering of Synchrotron Radiation, Part A, Hyperfine Interactions*, Vol. 123/124. Springer-Verlag.

[bb8] Gerdau, E. & de Waard, H. (2000). Editors. *Nuclear Resonant Scattering of Synchrotron Radiation, Part B, Hyperfine Interactions*, Vol. 125. Springer-Verlag.

[bb6] Gerdau, E., Rüffer, R., Winkler, H., Tolksdorf, W., Klages, C. & Hannon, J. P. (1985). *Phys. Rev. Lett.* **54**, 835–838. 10.1103/PhysRevLett.54.835 10031629

[bb9] Grote, M., Röhlsberger, R., Dimer, M., Gerdau, E., Hellmich, R., Hollatz, R., Jäschke, J., Lüken, E., Metge, J., Rüffer, R., Rüter, H. D., Sturhahn, W., Witthoff, E., Harsdorff, M., Pfützner, W., Chambers, M. & Hannon, J.-P. (1991). *Europhys. Lett.* **14**, 707–712.

[bb10] Haber, J., Schulze, K. S., Schlage, K., Loetzsch, R., Bocklage, L., Gurieva, T., Bernhardt, H., Wille, H.-C., Rüffer, R., Uschmann, I., Paulus, G. G. & Röhlsberger, R. (2016). *Nat. Photon.* **10**, 445–449.

[bb11] Hart, M. (1978). *Philos. Mag. B*, **38**, 41–56.

[bb12] Heeg, K. P., Haber, J., Schumacher, D., Bocklage, L., Wille, H.-C., Schulze, K. S., Loetzsch, R., Uschmann, I., Paulus, G. G., Rüffer, R., Röhlsberger, R. & Evers, J. (2015). *Phys. Rev. Lett.* **114**, 203601. 10.1103/PhysRevLett.114.203601 26047228

[bb13] Heeg, K. P., Wille, H.-C., Schlage, K., Guryeva, T., Schumacher, D., Uschmann, I., Schulze, K. S., Marx, B., Kämpfer, T., Paulus, G. G., Röhlsberger, R. & Evers, J. (2013). *Phys. Rev. Lett.* **111**, 073601. 10.1103/PhysRevLett.111.073601 23992063

[bb14] Kishimoto, S. (1992). *Rev. Sci. Instrum.* **63**, 824–827.

[bb15] Marx, B., Schulze, K., Uschmann, I., Kämpfer, T., Lötzsch, R., Wehrhan, O., Wagner, W., Detlefs, C., Roth, T., Härtwig, J., Förster, E., Stöhlker, T. & Paulus, G. G. (2013). *Phys. Rev. Lett.* **110**, 254801. 10.1103/PhysRevLett.110.254801 23829740

[bb16] Metge, J., Rüffer, R. & Gerdau, E. (1990). *Nucl. Instrum. Methods Phys. Res. A*, **292**, 187–190.

[bb17] Ramaseshan, S. & Ramachandran, G. (1954). *Proc. Indian Acad. Sci. A*, **39**, 20–30.

[bb18] Renninger, M. (1937). *Z. Phys.* **106**, 141–176.

[bb19] Röhlsberger, R. (2004). *Nuclear Condensed Matter Physics with Synchrotron Radiation – Basic Principles, Methodology and Applications*, No. 208 in *Springer Tracts in Modern Physics.* Springer-Verlag Berlin Heidelberg.

[bb20] Röhlsberger, R., Gerdau, E., Rüffer, R., Sturhahn, W., Toellner, T., Chumakov, A. & Alp, E. (1997). *Nucl. Instrum. Methods Phys. Res. A*, **394**, 251–255.

[bb22] Röhlsberger, R., Quast, K. W., Toellner, T. S., Lee, P. L., Sturhahn, W., Alp, E. E. & Burkel, E. (2001). *Phys. Rev. Lett.* **87**, 047601. 10.1103/PhysRevLett.87.047601 11461645

[bb23] Röhlsberger, R., Wille, H.-C., Schlage, K. & Sahoo, B. (2012). *Nature*, **482**, 199–203. 10.1038/nature10741 22318603

[bb24] Röhlsberger, R., Witthoff, E., Gerdau, E. & Lüken, E. (1993). *J. Appl. Phys.* **74**, 1933–1937.

[bb21] Röhlsberger, R. K. S., Schlage, K., Sahoo, B., Couet, S. & Rüffer, R. (2010). *Science*, **328**, 1248–1251. 10.1126/science.1187770 20466883

[bb25] Ruby, S. (1974). *J. Phys. Colloq.* **35**, C6-209–C6211.

[bb26] Schulze, K. (2018). *APL Photon.* **3**, 126106.

[bb27] Shvyd’ko, Y. (2013). *X-ray Optics: High-Energy-Resolution Applications*, Vol. 98 of *Springer Series in Optical Sciences.* Springer.

[bb28] Siddons, D., Hastings, J., Bergmann, U., Sette, F. & Krisch, M. (1995). *Nucl. Instrum. Methods Phys. Res. B*, **103**, 371–375.

[bb29] Sturhahn, W. (2000). *Hyperfine Interact.* **125**, 149–172.

[bb31] Toellner, T. S. (2000). *Hyperfine Interact.* **125**, 3–28.

[bb32] Toellner, T. S., Alp, E. E., Graber, T., Henning, R. W., Shastri, S. D., Shenoy, G. & Sturhahn, W. (2011). *J. Synchrotron Rad.* **18**, 183–188. 10.1107/S090904951003863X PMC3042327 21335904

[bb30] Toellner, T., Alp, E., Sturhahn, W., Mooney, T., Zhang, X., Ando, M., Yoda, Y. & Kikuta, S. (1995). *Appl. Phys. Lett.* **67**, 1993–1995. 10.1103/PhysRevLett.74.3832 10058308

[bb33] Trammell, G. T. & Hannon, J. P. (1978). *Phys. Rev. B*, **18**, 165–172.

